# Malocclusion in Early Anatomically Modern Human: A Reflection on the Etiology of Modern Dental Misalignment

**DOI:** 10.1371/journal.pone.0080771

**Published:** 2013-11-20

**Authors:** Rachel Sarig, Viviane Slon, Janan Abbas, Hila May, Nir Shpack, Alexander Dan Vardimon, Israel Hershkovitz

**Affiliations:** 1 Department of Orthodontics, Maurice and Gabriela Goldschleger School of Dental Medicine, Tel Aviv University, Tel-Aviv, Israel.; 2 Department of Anatomy and Anthropology, Sackler Faculty of Medicine, Tel-Aviv University, Tel-Aviv, Israel.; University of Kansas, United States of America

## Abstract

Malocclusions are common in modern populations. Yet, as the study of occlusion requires an almost intact dentition in both the maxilla and mandible, searching for the ultimate cause of malocclusion is a challenge: relatively little ancient material is available for research on occlusal states. The Qafzeh 9 skull is unique, as its preserved dentition allowed us to investigate the presence and manifestations of malocclusion. The aim of this study was thus to examine the occlusal condition in the Qafzeh 9 specimen in light of modern knowledge regarding the etiology of malocclusion. We revealed a pathologic occlusion in the Qafzeh 9 skull that probably originated in the early developmental stage of the dentition, and was aggravated by forces applied by mastication. When arch continuity is interrupted due to misalignment of teeth as in this case, force transmission is not equal on both sides, causing intra-arch outcomes such as mesialization of the teeth, midline deviation, rotations and the aggravation of crowding. All are evident in the Qafzeh 9 skull: the midline deviates to the left; the incisors rotate mesio-buccally; the left segment is constricted; the left first molar is buccally positioned and the left premolars palatally tilted. The inter-arch evaluation revealed anterior cross bite with functional shift that might affect force transmission and bite force. In conclusion, the findings of the current study suggest that malocclusion of developmental origin was already present in early anatomically modern humans (AMH) (the present case being the oldest known case, dated to ca. 100,000 years); that there is no basis to the notion that early AMH had a better adjustment between teeth and jaw size; and that jaw-teeth size discrepancy could be found in prehistoric populations and is not a recent phenomenon.

## Introduction

Malocclusion in general, and dental crowding in particular, are very rare findings among human fossils [1]. Several intrinsic and extrinsic factors (e.g., better adjustment between teeth and jaw size, different type and rate of dental attrition) have been proposed to explain the scarcity of these phenomena among our ancestors [2-4]. Nevertheless, since malocclusions can be examined only in well preserved skulls that have most of their teeth intact (to explore both the intra and inter-arch conditions) [5], evaluation of occlusal state is limited to very few fossils. It is therefore essential, when such an opportunity exists, to carry out a detailed orthodontic study in order to better understand our ancestors' masticatory system, by which we will be able to shed light on present day malocclusions. The Qafzeh 9 skull presents a well-preserved dentition of both the upper and lower jaws, allowing the exploration not only of the intra-arch condition, but also of inter-arch occlusion. 

Malocclusions are common in modern populations [6]. The most common condition is the anterior cross bite, found in 4-5% of the population, which usually develops at the early mixed-dentition stage [7-9]. In this condition, one or more primary or permanent mandibular incisors occlude labially against their antagonists, or one or more maxillary incisors are lingual to their antagonists [10]. Crowding is often ‘blamed’ for anterior cross bite [11], although other factors have been mentioned.

The etiology of cross bites (and Class I malocclusions in general) usually involves the initial position of the tooth buds and the environmental pressure that guides the eruption sequence [11]. Naturally, this pathology can occur only if the pressure lasts long enough to affect the displacement of the tooth buccally or palatally. Several factors have been suggested as the cause for anterior cross bite, including a lingual eruption path in the maxillary anterior incisors; trauma to the primary incisor resulting in lingual displacement of the permanent tooth germ; supernumerary anterior teeth; an over-retained necrotic or pulpless deciduous tooth or root; odontomas; crowding in the incisor region; inadequate arch length; and a habit of biting the upper lip [12-14].

There is a general agreement that anterior cross bite is often caused by modification in the masticatory function together with genetic or developmental components [11]. Abnormal occlusal interference (e.g. early tooth contact) caused by a constricted upper arch or a local factor such as a malposed tooth can result in mandibular displacement in centric occlusion. A mandible pushed laterally or anterior-posteriorly due to occlusal interferences can cause functional asymmetries, which in turn can prevent proper intercuspation in the centric relation [15]. 

The purpose of this study is to examine the occlusal condition in the Qafzeh 9 specimen in light of modern knowledge regarding the etiology of malocclusion. 

## Materials and Methods

### The Qafzeh 9 specimen

The Qafzeh Cave is located in the slope of Har Qedumim (Jebel Qafzeh) Lower Galilee, on the eastern bank of the Nahal Mizra (Wadi el-Haj) creek, in the Jezreel Valley, Israel. The dates for the site range between 94 ka to 115 ka [16-18]. 

The first excavations were conducted by R. Neuville and M. Stekelis in 1933-1935, during which the remains of seven individuals were uncovered in the Middle and Upper Palaeolithic layers. Excavations were renewed between 1965 and 1979 by B. Vandermeersch. During the excavations, the skeletal remains of several additional individuals, adults and immatures, were discovered in the Middle Palaeolithic layers [19,20]. 

The hominids found at Qafzeh were recognized as anatomically modern humans even though some primitive archaic features were present [19,21]. The occurrence of purposeful human burials, hearths, ochre and non-edible marine shells in the cave has been interpreted as evidence for the existence of a symbolic culture (see, among others, [19,22,23]. 

**Figure 1:**
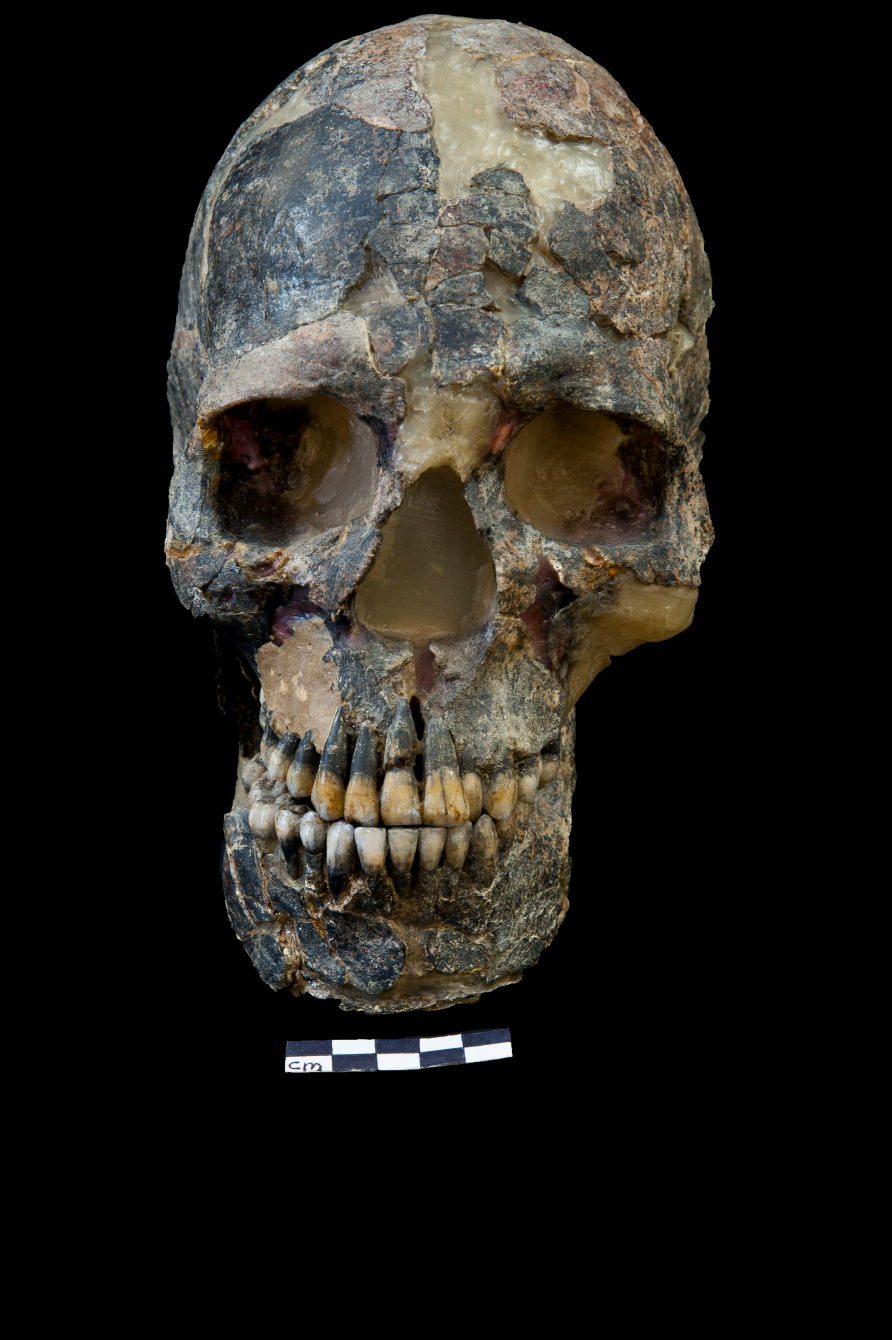
Frontal view of the Qafzeh 9 skull and mandible.

The skeleton and skull of Qafzeh 9, the subject of this paper (Figure 1), was found buried with the child Qafzeh 10, in the Mousterian layer XVII. Qafzeh 9 dental estimation exhibits open root apex of the third molar therefore, age of death was estimated to be between late adolescence and adulthood probably 16 and 21 years [24]. Two gender determinations were proposed based on pelvic study [19,25]. Recent analysis enhanced the assumption of female determination [24,26]. 

 Qafzeh 9 was found lying on its right side, in a semi-flexed position. The Qafzeh 9 skeleton is the most complete specimen found to date at the site [19]. 

A detailed osteological analysis of Qafzeh 9, with an emphasis on the cranium, was conducted by Vandermeersch [19]. Later studies on Qafzeh 9’s skeleton include, among others, analysis of the pelvis [25], femur [27], patella [28], hands [29], feet [30], mandible [31-33] and teeth [34,35].

The Qafzeh 9 specimen is housed in the Anthropological Collection at Tel-Aviv University. All necessary permits were obtained for the described study, which complied with all relevant regulations.

### Dental evaluation

The Qafzeh 9 skull was scanned using high resolution CT scans (iCT256, Philips Medical Systems, Cleveland, Ohio; slice thickness 0.67mm, voltage 120kV, current 298mA) taken at the Carmel Medical Center, Haifa, Israel. The scans were reviewed and analyzed using an “Extended Brilliance Workspace” portal (v2.6.0.27) (Philips Medical Systems, Cleveland, Ohio). 

The following aspects of the jaws were evaluated: teeth alignment, crowding, arch symmetry, occlusal attrition, non-occlusal attrition, occlusion and roots position. 

Teeth alignment was evaluated using Andrews' definition [36]. 

**Figure 2:**
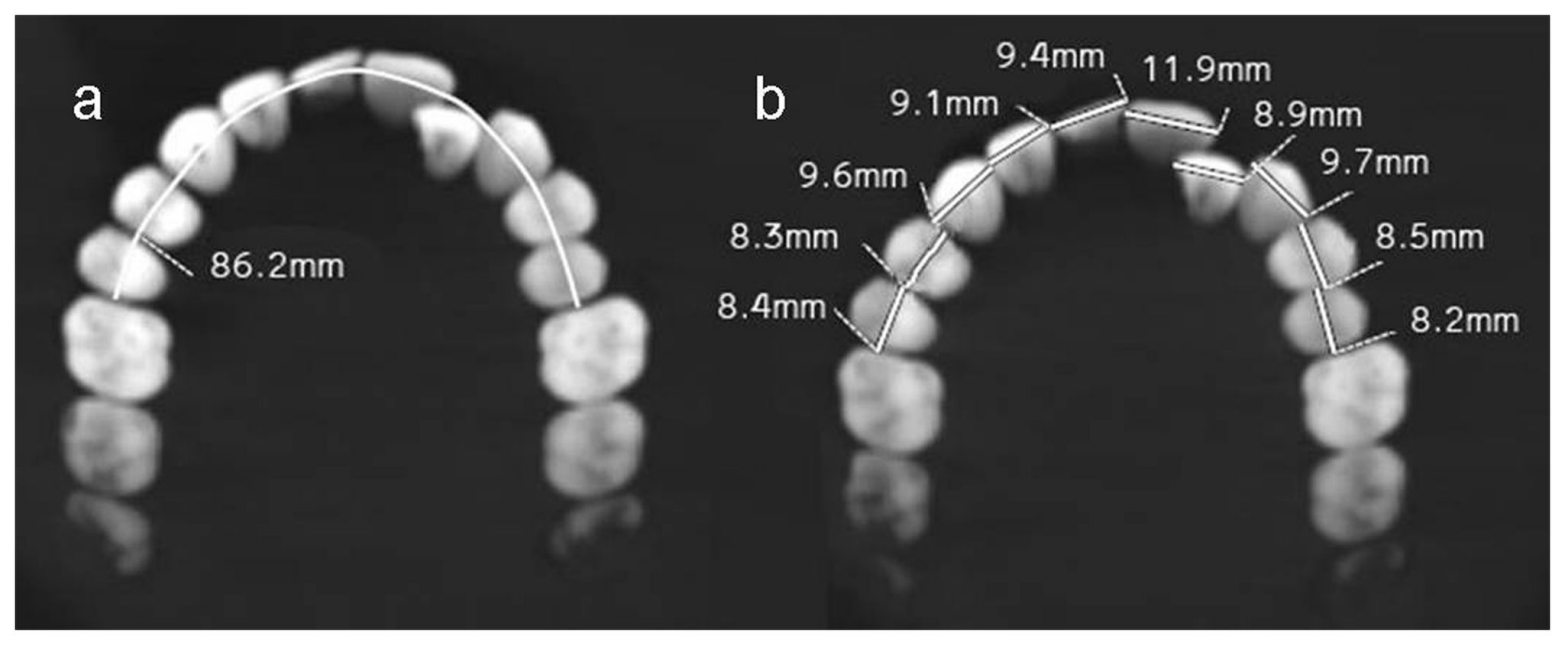
Evaluation of crowding. First, arch circumference (not including the molars) was measured along the contact points (a), then the sum of the mesial-distal sizes of the same teeth was measured (b). Crowding was calculated by subtracting arch circumference from the sum of all mesial-distal sizes of the teeth.

Dental crowding was evaluated on CT scans following Proffit method [11]: arch circumference (not including the molars) was measured along the contact points and subtracted from the mesial-distal size of the teeth (premolars, canines, and incisors) (Figure 2). 

**Figure 3:**
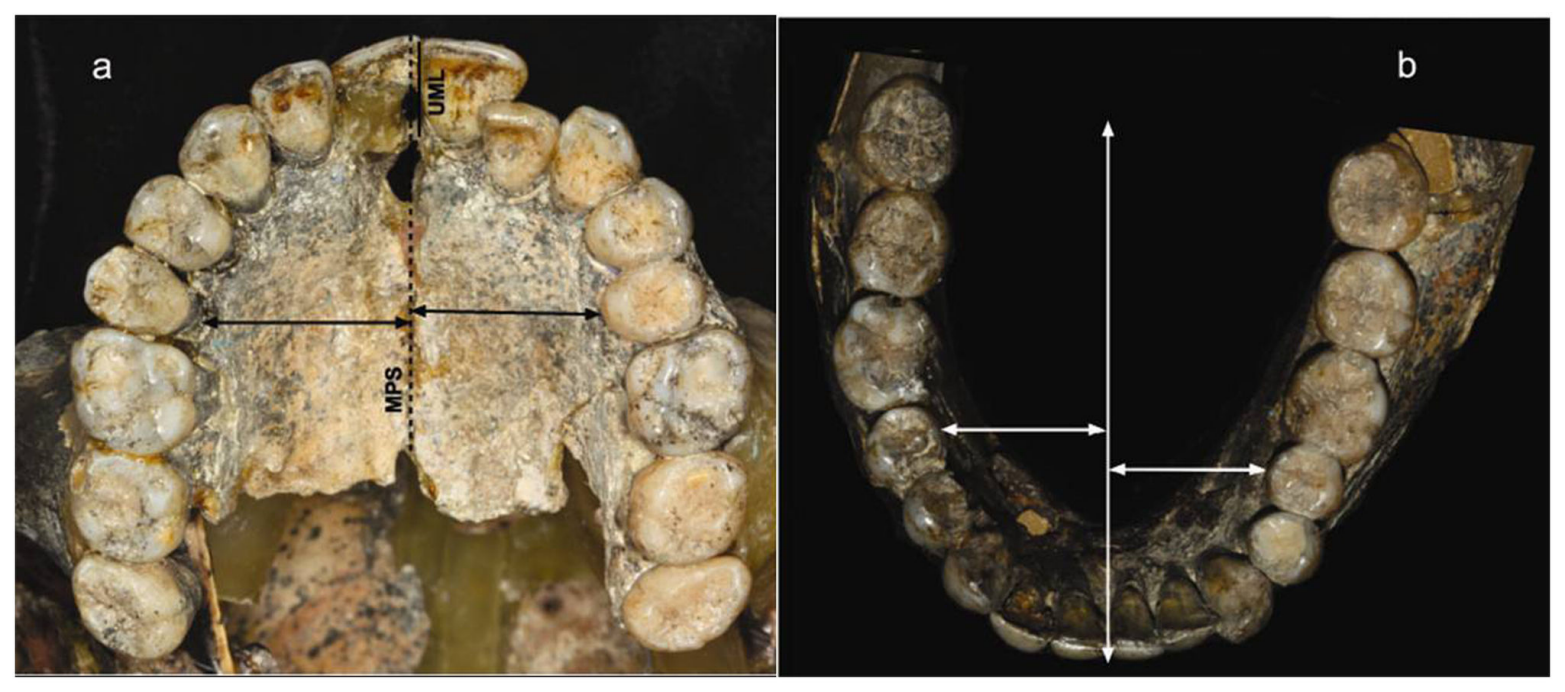
Occlusal view of the arches; crowding and asymmetry. (a) Occlusal view of the upper arch; (b) and of the lower arch. Note that upper arch symmetry and upper dental midline (UML) was evaluated relative to the palatal midline suture (MPS).

Arch symmetry was measured relatively to the mid-palatal suture (MPS) in the upper arch, and relatively to a midline drawn perpendicular to the central incisors in the lower arch (Figure 3).

Occlusal attrition rate was based on the Molnar scale [37]. Malocclusion was recognized following Andrews' definition [36]. Evaluation of occlusion status was problematic since the mandible could not be fitted to the maxilla properly, i.e., it was not possible to occlude the mandible in a manner that allows the condyles to seat in the fossa while matching the attrition facets. This could be due to post mortem changes or reconstruction difficulties both in the mandible and the skull. Therefore, a setup was used to evaluate occlusion in this skull. An impression of the lower arch was taken using a two-stage polyvinyl-siloxane (PVS) impression (Coltene Whaledent Germany). The cast was then created using dental stone material (orthodontic plaster type II, WhipMix, USA). The setup was carried out only for areas of the jaw where a previous reconstruction was carried out. The setup teeth were separated along the contact points and re-aligned to allow maximal intercuspation. The teeth of the setup were placed in maximal intercuspation. 

**Figure 4:**
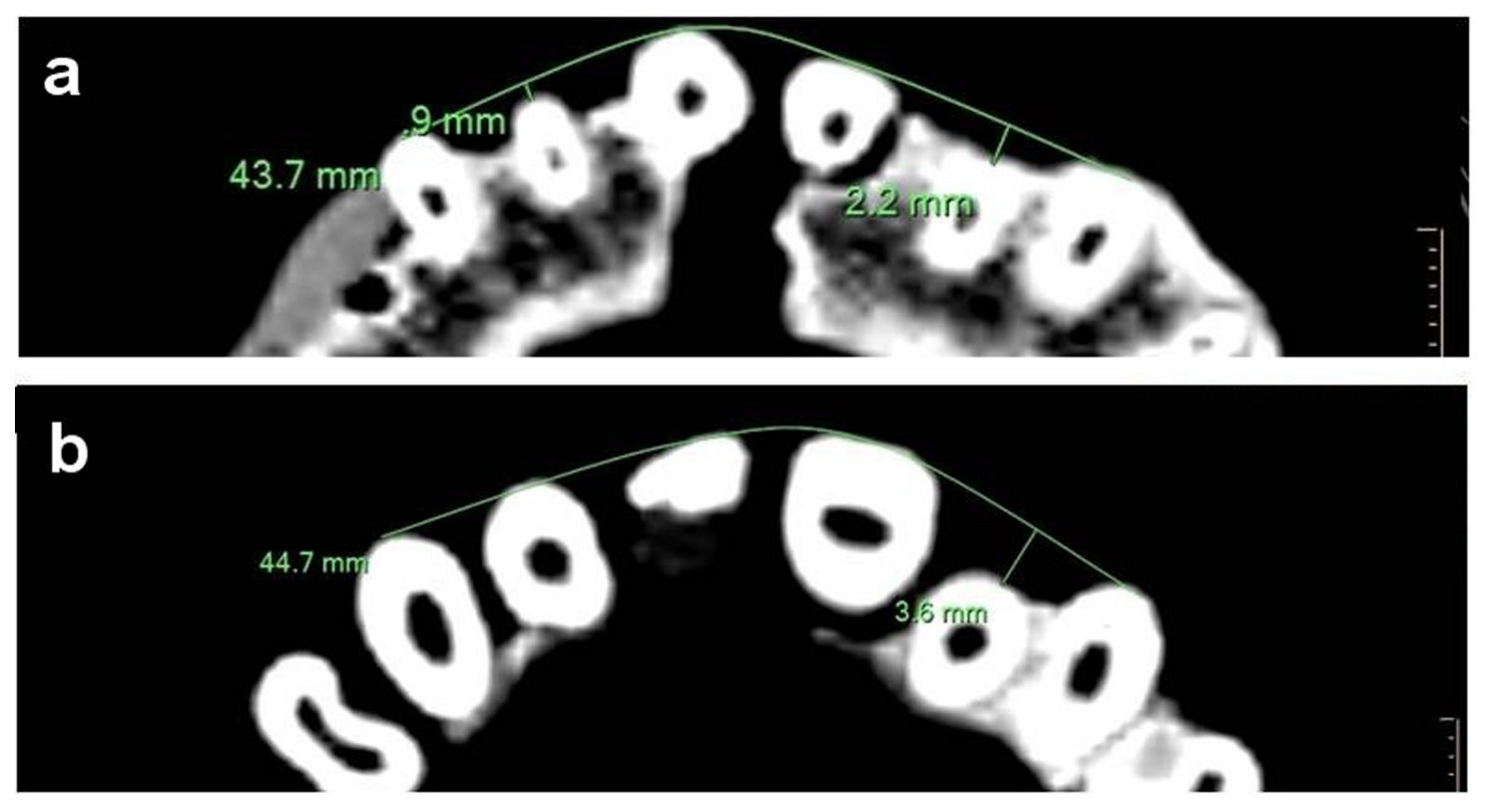
CT measurement of the position of the upper left lateral incisor. Measurements are shown in the apical (a) and gingival (b) segments.

Roots examination was carried out in order to appreciate the possibility of trauma. The roots of the upper incisors were examined (using CT scans) for the presence of fractures and root resorption. The position of the upper lateral incisors roots was measured as the distance from the midpoint marked on the buccal surface of the root to the line connecting the buccal midpoints of the canines and central incisors (Figure 4). This procedure was carried out for both the apical and the gingival segments.

## Results

### Dental alignment and crowding

From an occlusal view, the upper arch appears oval (Figure 3). The right central incisor is in mesio-buccal rotation. The upper left lateral incisor is palatally positioned (Figure 3). The left second premolar (PM) tilts palatally more than the adjacent molar. As a result, the contact point on the crown of the second PM is more buccally situated, whereas the one on the molar is positioned more palatally. The lower jaw arch corresponds to the oval shape of the upper jaw, its teeth are properly aligned. The left canine is slightly tilted buccally. 

The upper jaw's arch circumference is 86.2 mm, whereas the accumulated teeth's mesiodistal size is 92 mm, resulting in 5.8 mm of crowding (Figure 2). The mesiodistal diameter of the right central incisor (11.9 mm) is wider than the left (9.4 mm). The lack of interproximal attrition in the right central incisor left it with undisturbed morphology: a round distal margin with pronounced height of contour. 

### Arch symmetry

**Figure 5:**
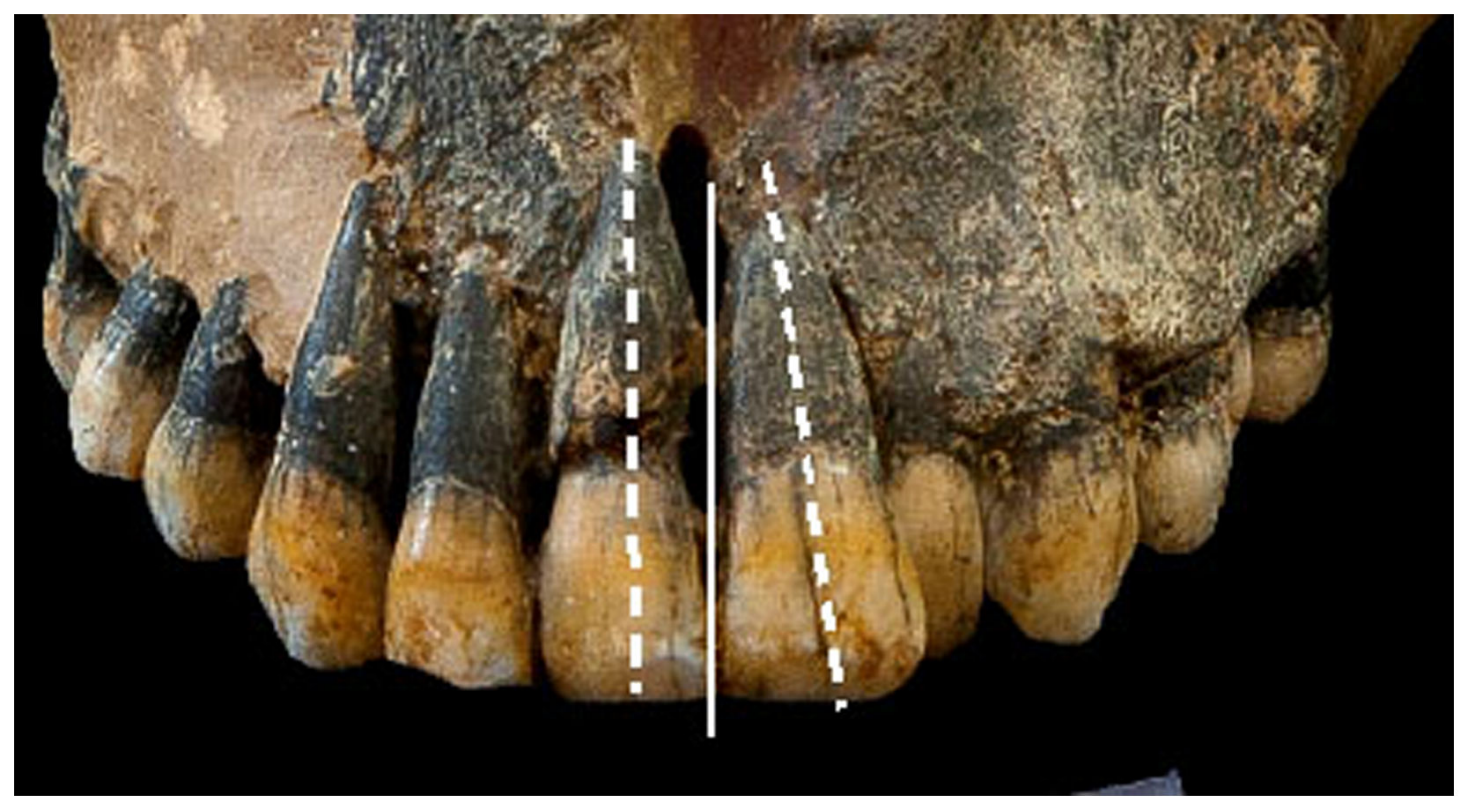
Frontal view of Qafzeh 9. Note the tilting of the left central incisor.

The breadth of the left half of the hard palate (measured at the second PM's level) was narrower than the right (Figure 3). The upper midline (UML) deviates (from the MPS) to the left by 2 mm (Figure 3). From a frontal view, the central incisors tilt towards the left (Figure 5). 

Although part of the lower arch asymmetry (Figure 3) is due to mal-reconstruction, a high degree of asymmetry is preserved on the setup. The deviation from the central line is more marked on the right side (Figure 3). This finding corresponds with the finding on the upper jaw.

### Occlusal and non-occlusal attrition

**Figure 6:**
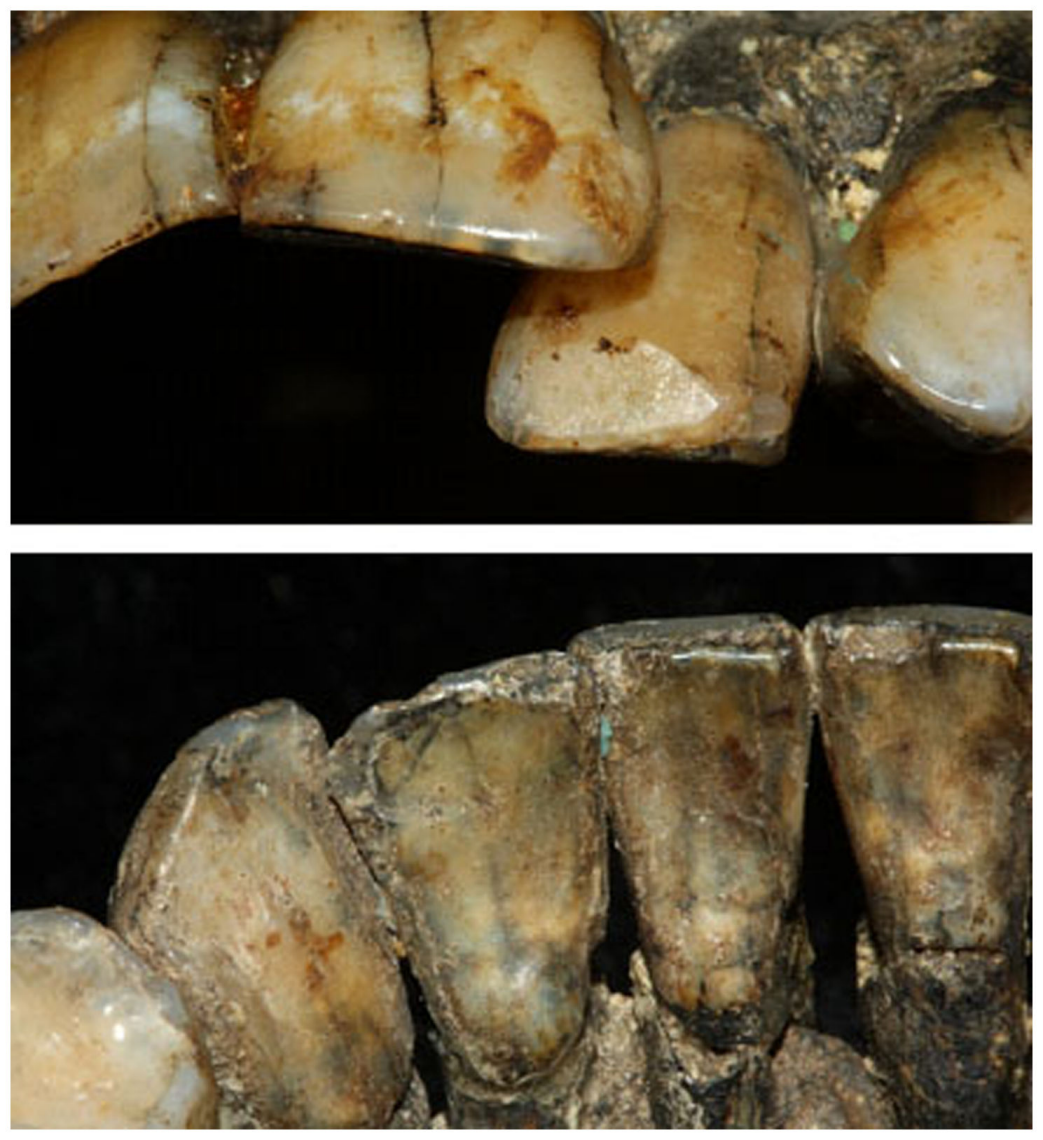
Non-occlusal attrition facets in Qafzeh 9. Note the buccal facet on the upper left lateral incisor (a) and the lingual facet on the lower left lateral incisor (b).

Occlusal attrition was slight (stage 1-2 on the Molnar scale), except for the upper and lower incisors where a patch of dentin was exposed (matching stage 3 on the Molnar scale). Beside the occlusal attrition facets, two other unique non-occlusal facets were observed: a buccal facet on the upper left lateral incisor and a lingual facet on the lower left lateral incisor (Figure 6). Interproximal attrition facets are evident in all teeth along the dental arch except for the distal surface of the upper left central incisor and mesial aspect of the upper left lateral incisor.

### Occlusion

**Figure 7:**
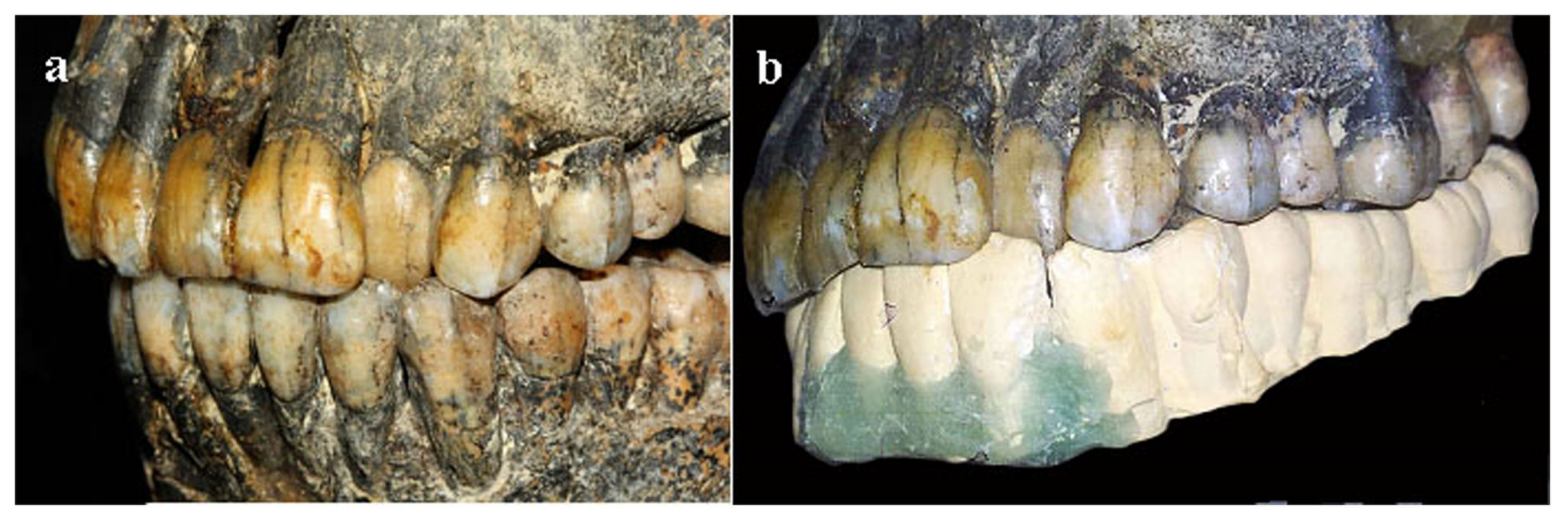
Anterior cross-bite in Qafzeh 9. An anterior cross bite caused by the malposed left lateral incisor (a). Following the setup, edge-to-edge contact with a tendency for a posterior cross bite in the buccal segments is clearly seen (b).

An anterior cross bite, caused by malposition of the upper left lateral incisor, is clearly seen (Figure 7). At the buccal segments (the area of molars and premolars), a shallow overbite and overjet, creating an edge-to-edge contact with a tendency for a posterior cross bite, was noticed. The palatal tipping of the left lateral incisor and the lack of contact with the antagonist lower incisor allowed the over eruption of the upper left lateral incisor. 

### Roots examination

 The root of the upper left lateral incisor is palatally positioned (Figure 4); the apical area of the root is located 2.2 mm palatally to the buccal margin of the arch, while on the right, it is only 0.9 mm (Figure 4). The gingival segment of the left tooth is distanced by 3.6 mm from the buccal margin, whereas on the right side, it reaches the buccal margin of the arch (Figure 4). 

## Discussion

Studying occlusion in fossils is a frustrating task, not only because of the rarity of appropriate material (complete maxilla and mandible), but also since even when the two jaws are present and all teeth are intact, in many cases the jaws are distorted. When articulating the Qafzeh 9 jaws, the incompatibility between the two is evident. This is due to a noticeable deformation in the lower jaw as a consequence of inadequate reconstruction as well as post-mortem taphonomical factors (see also 33). The use of a cast setup allowed us to fix areas that were inadequately reconstructed in the past and restore the original shape of the lower dental arch. Once this was done, the teeth were placed in maximal intercuspation with attrition facets taken into account, which allowed the evaluation of the occlusion condition [38]. 

The most noticeable deviation from normal occlusion was the malposition of the upper left lateral incisor. It is difficult to evaluate what was the direct cause of the malposed upper left lateral incisor, as many factors might cause malposition of a single tooth. Nevertheless, trauma can be excluded since following trauma, we would expect the crown to tip palatally while the root keeps its position (or even tilts buccally). In our case, however, the root of the upper left lateral incisor is located palatally to the dental arch, indicating that the tooth position had already been established during early life (development and eruption stages). The absence of an interproximal facet on the distal surface of the upper central incisor and on the mesial surface of the lateral incisor indicates that the two teeth were never in contact. This lends additional support to our suggestion that this malocclusion did not result from a traumatic event, but rather is of developmental origin. 

**Figure 8:**
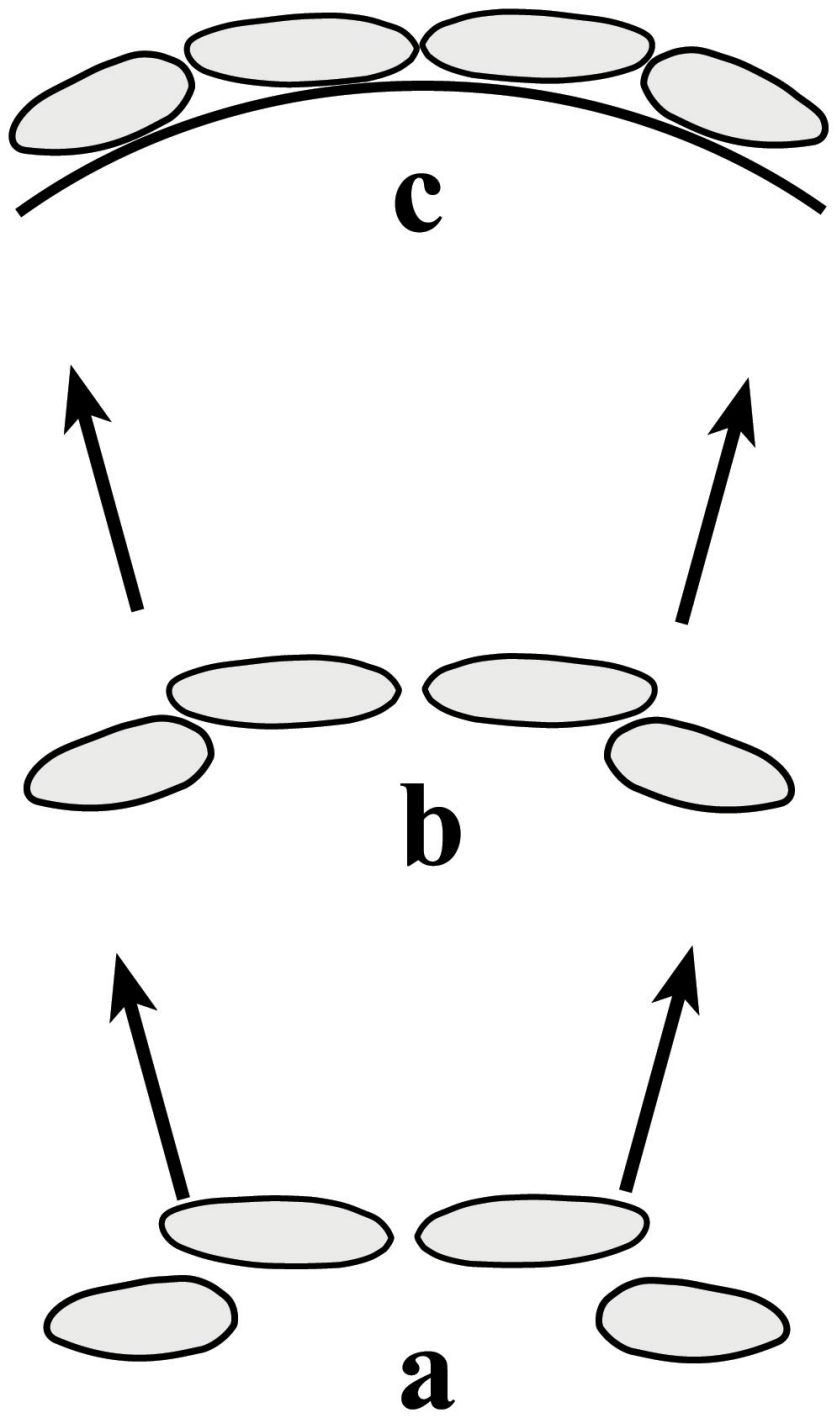
Development of the anterior dental arch. Lateral incisors buds are formed more palatally compared to the central incisors (a) and erupt more anteriorly (b) to finally align with the central incisors (c).

Relative to central incisors, lateral incisor buds are formed in a more palatal position (Figure 8a). During eruption, the lateral incisors move buccally (Figure 8b) to align with the central incisors (Figure 8c). However, early loss of deciduous tooth or crowding in the upper jaw may interfere with the normal developmental process described above and cause malposition of teeth similar to that seen in Qafzeh 9. Once this occurs, the entire biomechanical force transmission, both in the intra and inter-arch, is affected, with noticeable morphological consequences. 

**Figure 9:**
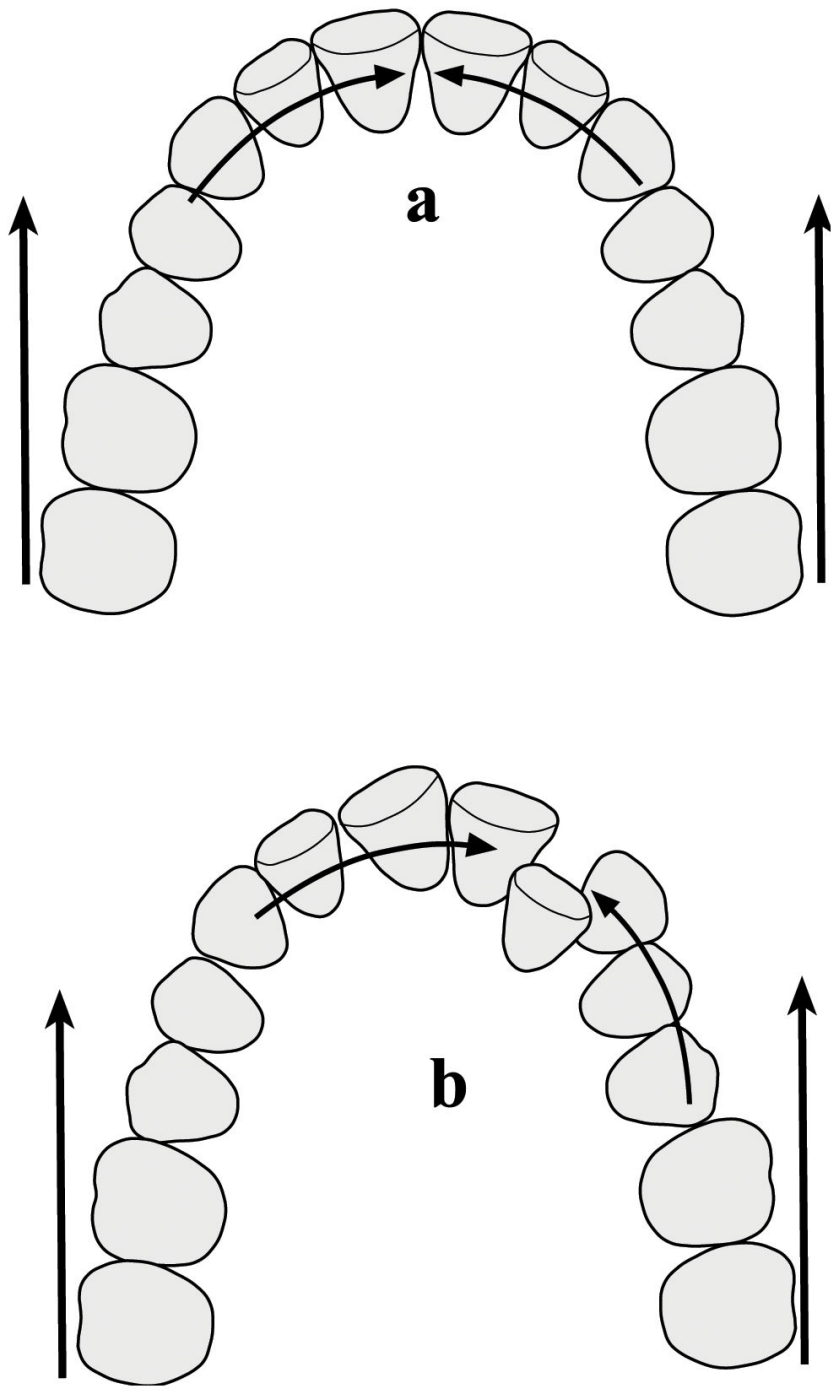
Force transmission in the dental arch. When arch integrity is preserved, there is similar dissipation of force on both sides maintaining arch symmetry (a). When arch continuity is interrupted (like in the Qafzeh 9 skull), force transmission is not equal on both sides (b), resulting in asymmetry, crowding, midline deviation and rotations.

Regarding the intra-arch effect, physiologically, contact between teeth lessen the masticatory forces along the dental arch [39-41], thus preventing mesial migration of teeth [42], protecting arch integrity and avoiding food impaction [43]. The occlusal forces applied to the arch are also transformed into interproximal forces and interproximal attrition. When arch integrity is preserved, arch symmetry is kept allowing similar dissipation of force on both sides (Figure 9a). When arch continuity is interrupted, force transmission is not equal on both sides (Figure 9b). The anterior component of the force caused by the occlusal forces may result in mesialization of the teeth, midline deviation, rotations and the aggravation of crowding [44]. All these potential outcomes are evident in the Qafzeh 9 skull: the midline deviates to the left; the incisors rotate mesio-buccally; the left segment is constricted; the left first molar is buccally positioned and the left premolars palatally tilted. 

As to the inter-arch effect, when the jaws are in occlusion, the palatal position of the left lateral incisor causes the mandible to occlude in a more anterior and lateral position, resulting in a cross bite with a functional shift. This is evidenced by the buccal facet found on the maxillary left lateral incisor and the lingual facet found on the mandibular left lateral incisor of Qafzeh 9. This type of occlusion may lock the mandible in a position that does not coincide with the centric occlusion expected in this individual. 

Cross bite might cause asymmetrical muscle function during chewing or clenching, as the temporalis muscle is more active and the masseter muscle less active on the cross bite side than on the non-cross bite side [45,46]. Moreover, the asymmetry in muscle activity that is associated with the cross bite might reduce the bite force [47,48]. 

The notion that ancient populations had better aligned dentitions than modern ones is well rooted in the anthropological and dental literature (e.g., 2,3,11,49,50). Most of the evidence was obtained from orthodontic studies carried on historical (mainly Medieval populations) or modern pre-industrial populations demonstrating low prevalence of malocclusion compared to modern populations (e.g.,[51-55]). Yet, not just that the prevalence was lower, but the severity was smaller and there was a significant sex-biased towards females [56]. It is of note worthy, however, that the above described trends have been shown for specific populations (mainly Europeans), that the time depth is limited (several hundred years), that modern populations varies in regard to malocclusion prevalence [57], and that the relative contributions of heredity and environment to the etiology of malocclusion varies among its different entities [51]. As the great obstacle in studying trends in malocclusion remained the small sample size of prehistoric skulls suitable for such studies, Vodanović and colleagues [5] have recently suggested to move from orthodontic features requiring presence of both jaws and almost all teeth to orthodontic anomalies affecting only one tooth or group of teeth. Finally, it was suggested (e.g., 58,59) that dental crowding is a result of an evolutionary trend towards a reduced jaw size, without a corresponding reduction in tooth dimension a process usually attributed to reduction in masticatory requirements due to nutritional change, i.e., softer food [60]. However, the study of the Qafzeh 9 jaws together with previous findings from Neanderthals and Upper Paleolithic skulls [61] may suggest a more complex etiology for malocclusion (for further discussion see 52,62). 

In sum, the well-preserved dentition in the Qafzeh 9 skull allowed us also to orthodontically evaluate its inter and intra-arch relationships. The presence of a clear malocclusion of developmental origin in this specimen is the oldest recorded in the hominid lineage. The upper crowding and the malposed teeth affect not only the inter-arch dental alignment, but also the intra-arch association, causing an anterior cross bite with functional shift, all of which may affect the bite force and the force transmission along the dental arch. 

## Conclusions

The analysis of the Qafzeh 9 jaws and teeth clearly show that crowding and malocclusion are present in early anatomically modern humans. These findings challenge the notion that early anatomically modern humans had a better adjustment between teeth and jaw, and question the common theories for crowding, suggesting an increase in jaw-teeth size discrepancy towards modern times. It also questions the role of soft diet and may indirectly indicate that genetic may prevails over environment. 
